# m6A-related lncRNAs predict prognosis and indicate cell cycle in gastric cancer

**DOI:** 10.3389/fgene.2023.1140218

**Published:** 2023-06-20

**Authors:** Dong Wan, Lingnan He, Cheng Guo, Zishao Zhong, Xiaohan Yan, Jia Cao, Qinwei Xu, Haibin Zhang, Bensong Duan

**Affiliations:** ^1^ Endoscopy Center, Shanghai East Hospital, Tongji University School of Medicine, Shanghai, China; ^2^ Guangdong Provincial Hospital of Chinese Medicine, Guangzhou, China

**Keywords:** N6-methyladenosine, gastric cancer, long non-coding RNA, prognosis, cell cycle

## Abstract

**Background:** N6-methyladenosine (m6A) modification is a common epigenetic methylation modification of RNA, which plays an important role in gastric carcinogenesis and progression by regulating long non-coding RNA (lncRNA). This study is aimed to investigate the potential prognostic signatures of m6A -related lncRNAs in STAD.

**Methods:** The m6A-related lncRNAs with the most significant impact on gastric cancer prognosis in the TCGA database were identified by bioinformatics and machine learning methods. The m6A-related lncRNA prognostic model (m6A-LPS) and nomogram was constructed by Cox regression analysis with the minimum absolute contraction and selection operator (LASSO) algorithm. The functional enrichment analysis of m6A-related lncRNAs was also investigated. The miRTarBase, miRDB and TargetScan databases were utilized to establish a prognosis-related network of competing endogenous RNA (ceRNA) by bioinformatics methods. The correlation of AL391152.1 expressions and cell cycle were experimentally testified by qRT-PCR and flow cytometry.

**Results:** In total, 697 lncRNAs that were identified as m6A-related lncRNAs in GC samples. The survival analysis showed that 18 lncRNAs demonstrated prognostic values. A risk model with 11 lncRNAs was established by Lasso Cox regression, and can predict the prognosis of GC patients. Cox regression analysis and ROC curve indicated that this lncRNA prediction model was an independent risk factor for survival rates. Functional enrichment analysis and ceRNA network revealed that the nomogram was notably associated with cell cycle. qRT-PCR and flow cytometry revealed that downregulation of GC m6A-related lncRNA AL391152.1 could decrease cyclins expression in SGC7901 cells.

**Conclusion:** A m6A-related lncRNAs prognostic model was established in this study, which can be applied to predict prognosis and cell cycle in gastric cancer.

## Introduction

Gastric cancer is the fifth most prevalent malignant tumor and the third most deadly tumor in the world, causing about 800,000 deaths each year and seriously threatening human life and health (Sung et al., 2021). Currently, treatment options remain limited due to the heterogeneity of GC and the unclear mechanisms of development and progression. Moreover, the high recurrence rate after GC resection in advanced stage of GC and the tendency of chemotherapy resistance in case of recurrence make for a poor prognosis, and the median survival period for stage IV GC is only about 9–10 months (Ajani et al., 2016; Sasahara et al., 2021). GC urgently needs more in-depth research on its pathogenesis and therapeutic targets.

N6-methyladenosine (m6A) modifications are common epigenetic methylation modifications of messenger RNAs (mRNAs) and non-coding RNAs (ncRNAs) (Zhao et al., 2017; Dai et al., 2018). The m6A modification occurs mainly clustered on the adenine in the sequence of RRACH (R = G or A, H = A, C or U) of the stop codon, 3′untranslated region (3′UTR), and inner ministerial exon (Meyer et al., 2012; Linder et al., 2015; Zhang H. et al., 2020). There are three well-known types of regulators that dynamically control m6A: “Writers”, “Readers”, and “Erasers”. METTL3, METTL14, METTL16, and WTAP, *etc.*, have been confirmed to be “Writers” genes, FTO and ALKBH5 are “Erasers” genes, and YTHDC1 and YTHDC2, *etc.*, are “Readers” genes. RNA is methylated by the effect of “Writers” and demethylated by the effect of “Erasers”. The m6A-modified RNA is recognized by “Readers” and affects RNA processing, nuclear export, translation, decay, *etc.,* (Zhang H. et al., 2020). This dynamic, reversible biological process regulates development, progression and immune response of GC. For example, upregulated m6A “Writers” METTL3, WTAP and RBM15 mediated poor prognosis of GC by regulating m6A, and upregulated “Erasers” ALKBH5 mediated poor prognosis of GC by regulating the m6A/lncRNA NEAT1/EZH2 axis (Zhang J. et al., 2019; He et al., 2019; Su et al., 2019; Yue et al., 2019; Zhu et al., 2019; Wang et al., 2020; Yang et al., 2020).

Long non-coding RNA (lncRNA) plays an important role in the ontogenesis and development of GC. LncRNAs influence gastric carcinogenesis and progression through mechanisms such as histone modification, DNA methylation, hydroxymethylation, chromatin remodeling, acting as a molecular sponge for miRNAs, and direct protein binding (Bayoumi et al., 2016). However, the mechanism of how m6A modifications affect the dysregulation of lncRNAs causing gastric carcinogenesis and progression remains unclear. Therefore, understanding the lncRNAs related to m6A modifications involved in GC progression may help to identify useful therapeutic targets.

In this study, we investigated GC data from TCGA database by bioinformatics and machine learning methods to find m6A-related lncRNAs with the greatest impact on GC prognosis, and constructed m6A-related lncRNAs prognostic signatures (m6A-LPS), and nomogram based on m6A-LPS and clinicopathological features. In our study, we found that m6A-LPS performed well in predicting overall survival in the TCGA GC dataset, and downregulation of GC m6A-related lncRNA AL391152.1 could decrease cyclins expression in SGC7901 cells.

## Materials and methods

### Data sources

Fragments per kilobases per million (FPKM) of RNA Sequencing data for GC patients were downloaded from the TCGA GDC website (https://portal.gdc.cancer.gov/). The long non-coding RNA were annotated by Genome Reference Consortium Human Build 38 (GRCh38) was acquired from the GENCODE website (https://www.gencodegenes.org/human/).

### The m6A-related and prognosis-related lncRNA identification

Differential expression using the limma package in R for the identification of GC-related lncRNAs (Ritchie et al., 2015). Pearson correlation analysis was performed for identifying of m6A-related lncRNAs with the | Pearson R| > 0.5 and *p*-value <0.001. The m6A-related lncRNAs for GC was the intersection of GC-related lncRNAs and m6A-related lncRNAs.

The association between expression of m6A-related lncRNAs for GC and overall survival was evaluated by univariate Cox regression analysis. The m6A-related lncRNAs for GC with *p* values < 0.05 were considered as candidate m6A-related prognostic lncRNAs for GC.

### Construction of m6A-related lncRNA prognostic model

Dataset was randomly splited into a training set and a test set at a ratio of 1:1. Training set was used for training model, and test set was used for evaluating model performance. A LASSO Cox regression model was constructed to select the most significant prognostic markers among the candidate m6A-related prognostic lncRNAs for GC. Multivariate Cox regression calculate the coefficient of each lncRNA selected above. A prognostic risk score was calculated by each expression values of the lncRNAs and their corresponding estimated regression coefficients as the following formula:

$$Risk\;score=\sum_{i=1}^{n}{Coefficient}_{i}\times {expression\;value}_{i}$$

Coefficient_i_ was the regression coefficient in LASSO regression and expression value_i_ was the expression value of each lncRNAs. The high- and low-risk score groups are divided by the median risk score of the training set as the threshold. Kaplan-Meier survival curve test and log-rank test were performed to investigate survival differences between high- and low-risk score groups and between subgroups with various clinicopathological characteristics.

### M6A-related lncRNA prognostic nomogram construction

The univariate and multivariate COX regression analysis with clinical characteristics and the risk scores constructed above was performed to understand the independent risk factors associated with prognosis. The “rms” package (http://hbiostat.org/R/rms) was used to build the prognostic nomogram. The performance of the nomogram for overall survival prediction was evaluated with AUC values calculated from ROC curves, and calibration curves.

### Functional enrichment analysis

Differential gene expression analysis for high and low risk score groups was performed with the limma package (Ritchie et al., 2015). For differential genes meeting the criteria (*p* < 0.05, |logFC| > 1), Gene ontology (GO) and Kyoto Encyclopedia of Genes and Genomes (KEGG) signaling pathway enrichment analysis was performed and plotted with the clusterProfiler package (Yu et al., 2012).

### Cell lines and cell culture

The human gastric cancer cell line SGC7901 were purchased from American Type Culture Collection (ATCC). GC cell lines were identified by short tandem repeat analysis, and the results of *mycoplasma* test were negative and were cultured with RPMI-1640 medium (Gibco, San Francisco, CA, United States) containing 10% fetal bovine serum (Gibco) with 100 U/mL penicillin and streptomycin (Gibco, Shanghai, China) at 37°C in a humidified incubator of 5% CO2.

### siRNA construction and infection

siRNA sequences targeting lncRNA AL391152.1 was designed and constructed by Obio Technology Co. Ltd., Shanghai, China. siRNA was added to the culture medium of SGC7901 cells by using riboFECT Transfection Kit (Ribobio, Shanghai, China). The targeted AL391152.1 sequences were as follows: #1 GGC​CCT​AGA​GAG​CAT​TAT​ATC,#2 GGT​GTT​TAA​CAG​ATA​GCA​TTG.

### RNA extraction and qRT-PCR

Total RNA was extracted from samples with RNAi Plus reagent (TAKARA, Japan) and quantified by using Nanodrop 8000 and stored at −80°C. 1,000 ng of RNA was reversely transcribed into cDNA using a reverse transcription system (TAKARA, Japan). Real-Time qPCR was performed to quantify the transcripts using TB Green PCR Master Mix (TAKARA, Japan). The amplification primers for the AL391152.1 coding region was as follows: F: ACC​TAA​ATC​TTT​TCT​CAC​TCA​CTT​TT, R: TGG​AAG​CAA​CGA​TTA​AGC​AAA​ACT. The relative abundance of RNA was normalized to β-Actin.

### Flow cytometry

Cell cycle of SGC7901 was evaluated by flow cytometry using a Cell Cycle Detection Kit (keyGEN bioTECH, Nanjing, China) according to the manufacturer’s protocol. Briefly, the SGC7901 cells were collected and washed with cool PBS, gently resuspended and incubated with Propidium Iodide (PI) in the dark for 15 min and analyzed by flow cytometry.

### Statistical analysis

Gene expression correlations were analyzed by Pearson correlation analysis. Gene expression or risk scores between groups were compared by analysis of variance (ANOVA). Overall survival differences between subgroups were examined by Kaplan-Meier curves and log-rank tests. The performance of predictors at each time point was estimated with receiver operating characteristic (ROC) curves and area under the curve (AUC) values. Statistics were performed with R software (version 4.00). *p*-values less than 0.05 were considered statistically different.

## Results

### Identification of m6A-related LncRNA in GC patients

**FIGURE 1:**
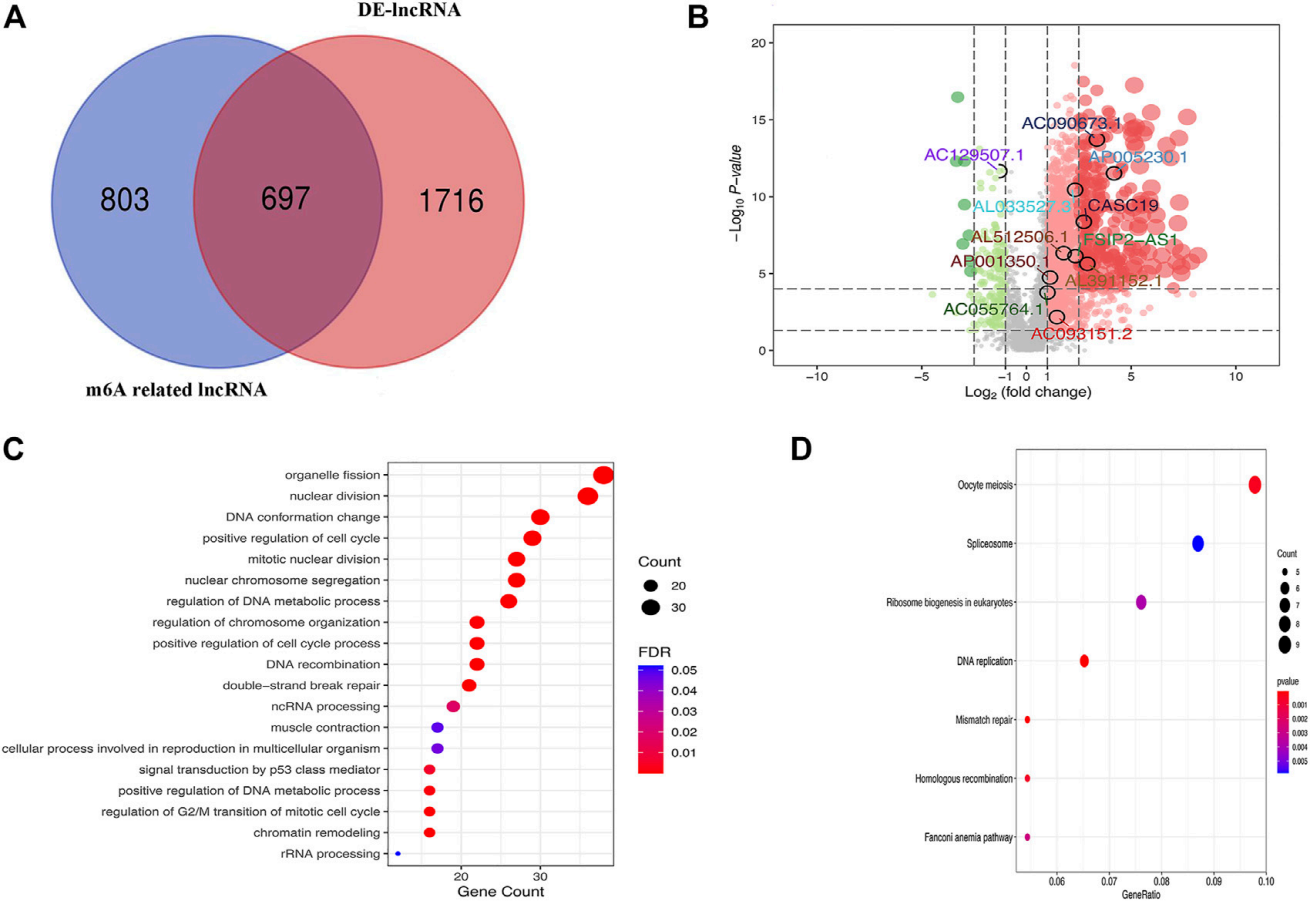
**(A)** Venn diagram of the counts of m6A-related lncRNAs and differentially expressed lncRNAs between GC and paracancer (DE-lncRNA); **(B)** Volcano map with labeled prognostic GC m6A-related lncRNAs; **(C)** GO analysis of m6A-related lncRNAs; **(D)** KEGG analysis of m6A-related lncRNAs.

The 380 tumor samples and 27 paracancerous samples of GC patients from the TCGA database were included. GRCh38 from GENCODE for identification of genes as lncRNAs. A total of 2413 GC and paracancer differential LncRNAs and 1,500 m6A-related lncRNAs were obtained. The intersection had a total of 697 lncRNAs that were identified as m6A-related lncRNAs (Figure 1A). Survival analysis was performed on 697 GC m6A-related lncRNAs, and a total of 18 lncRNAs were identified as associated with overall survival, which were LINC02428, LINC00106, FSIP2-AS1, DKFZp779M0652, CASC19, AP005230.1, AP001350.1, AL807757.2, AL513123.1, AL512506.1, AL391152.1, AL033527.3, AL031123.2, AC129507.1, AC093151.2, AC090673.1, AC090192.2, and AC05764.1. A volcano map with labeled prognostic GC m6A-related lncRNAs was shown in Figure 1B. GO and KEGG analysis of m6A-related lncRNAs were shown in Figures 1C, D.

### Construction of GC m6A-related LncRNA prognostic model

**FIGURE 2:**
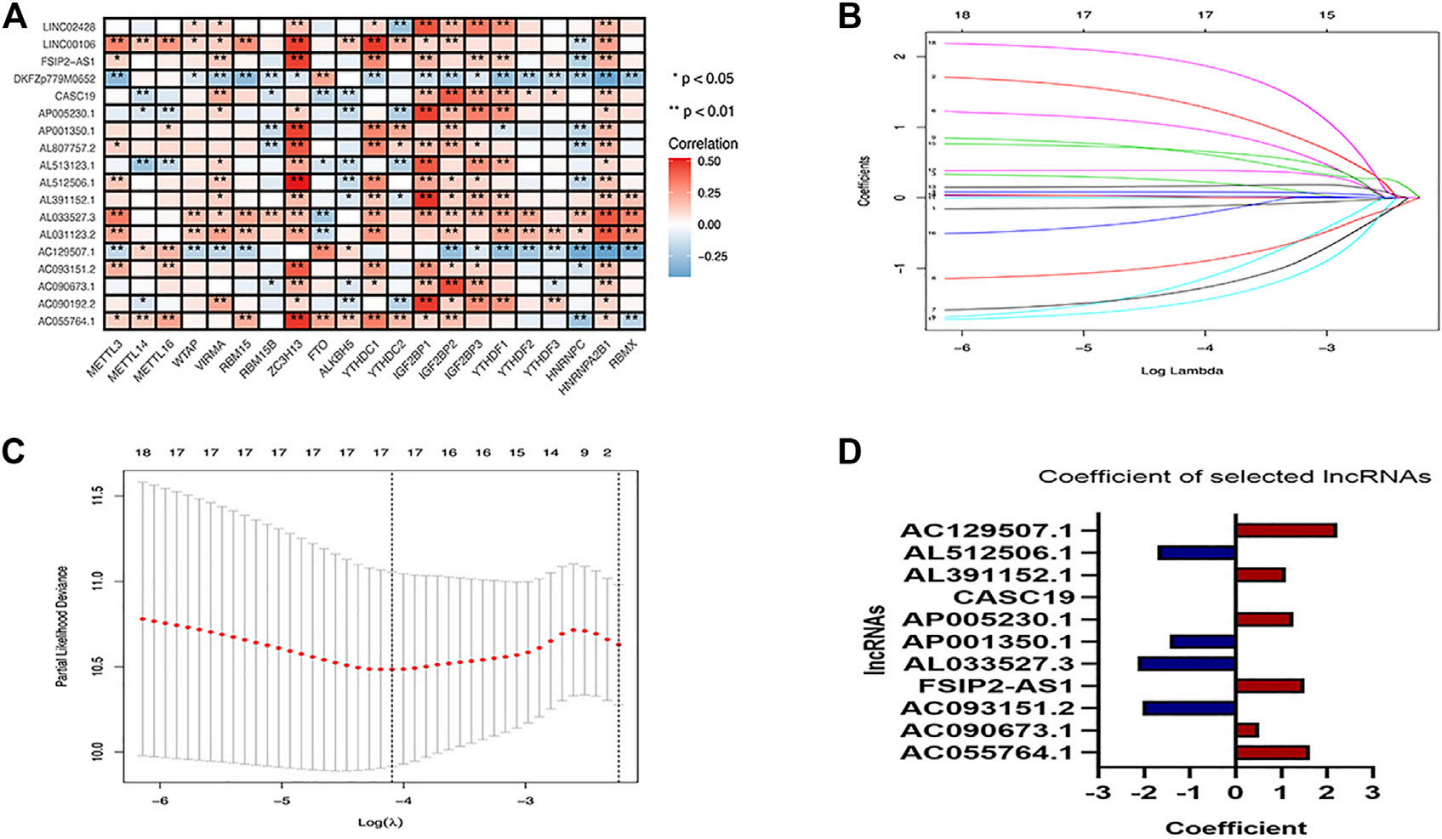
**(A)**Heatmap of the correlation between m6A-related genes and 18 prognostic m6A-related lncRNAs; **(B,C)** LASSO regression compresses 18 prognostic m6A-associated lncRNAs into 11; **(D)** Coefficients of each lncRNA selected by LASSO regression in the regression model.

**FIGURE 3:**
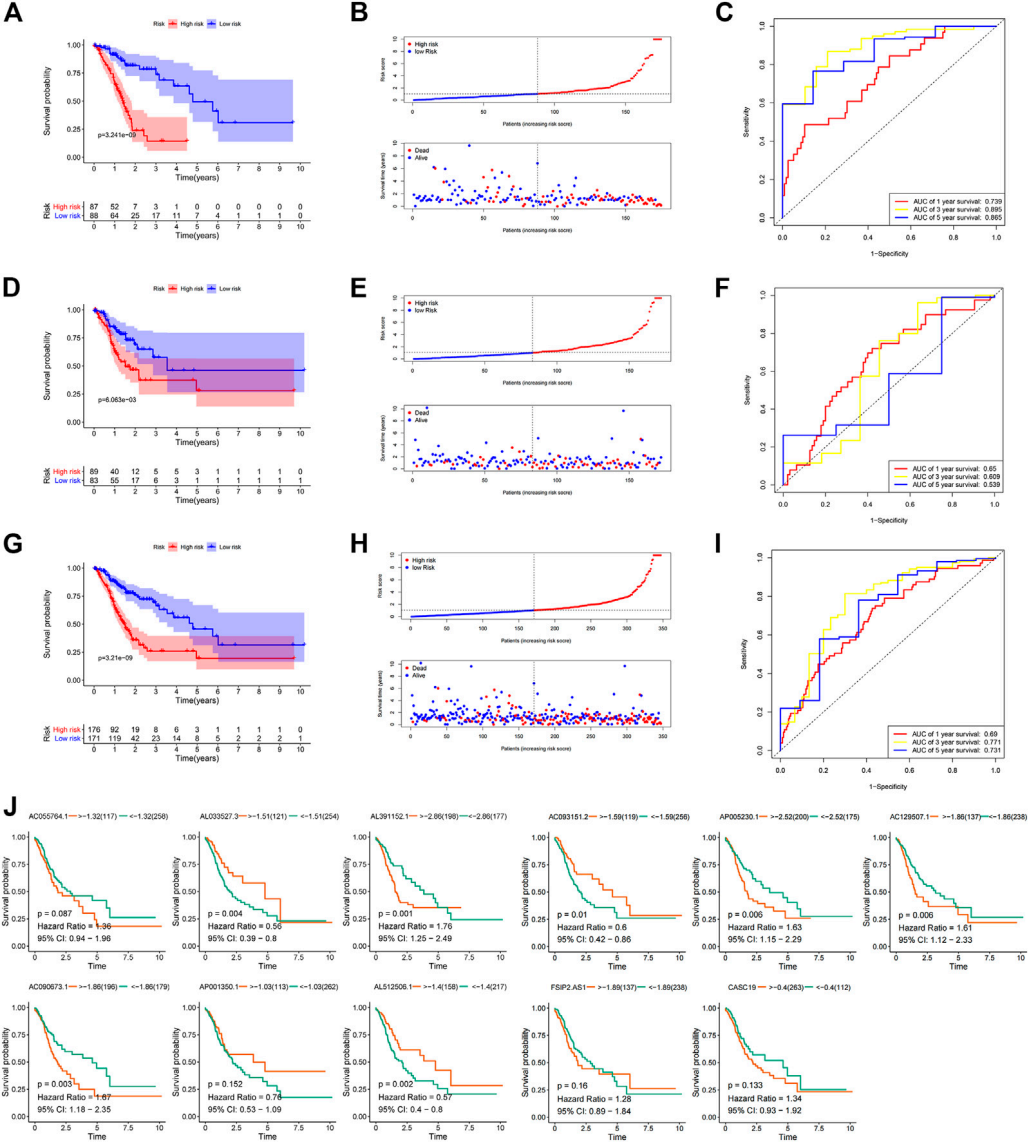
**(A)** Kaplan-Meier survival curves for the high and low risk score groups of the training set; **(B)** Survival distribution of high and low risk score groups in the training set; **(C)** ROC curve of m6A-LPS for predicting 1/3/5-year overall survival of GC patients in the training set; **(D)** Kaplan-Meier survival curves for the high and low risk score groups of the test set; **(E)** Survival distribution of high and low risk score groups in the test set; **(F)** ROC curve of m6A-LPS for predicting 1/3/5-year overall survival of GC patients in the test set; **(G)** Kaplan-Meier survival curves for the high and low risk score groups of the full set; **(H)** Survival distribution of high and low risk score groups in the full-set; **(I)** ROC curve of m6A-LPS for predicting 1/3/5-year overall survival of GC patients in the full set; **(J)** Kaplan-Meier survival curves for each lncRNA in m6A-LPS in the TCGA GC dataset, with the median expression as the grouping cut-off.

**FIGURE 4:**
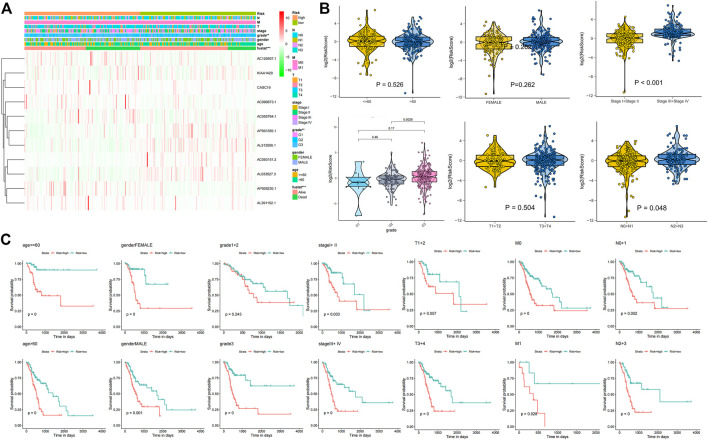
**(A)** Heatmap of differentially expressed genes between the high and low risk score groups along with clinicopathological subgroups. **(B)** The difference of age, gender, stage, grade, T, and N in high and low risk groups; **(C)** Kaplan-Meier survival curves for high and low risk score groups in each clinicopathologic features subgroup of the TCGA GC dataset.

The correlation of the 18 GC m6A-related prognostic lncRNAs with each of the m6A genes is shown in Figure 2A. To construct GC m6A-related lncRNA prognostic models, GC samples from the TCGA database were randomly divided into a training set and a test set by 1:1. The training set is for constructing the model, while the test set is for evaluating the model. Eighteen candidate GC m6A-related prognostic lncRNAs were applied for LASSO regression analysis. The final 11 m6A-related prognostic lncRNAs were enrolled in the prognostic model and defined as m6A-related lncRNA prognostic signatures (m6A-LPS) (Figures 2B, C). The risk score was the sum of the expression values of each lncRNA multiplied by their coefficient (Figure 2D). As demonstrated in Figures 3A, D, G, overall survival of patients with high and low m6A-LPS risk scores was significantly different in the training set, test set, and full set (*p* < 0.05). The survival distributions of high and low risk scores in the training set, test set, and full set are shown in Figures 3B, E, H. The predictive performance of m6A-LPS for overall survival of GC patients at 1, 3, and 5 years had AUC values of 0.739, 0.895, and 0.865 in the training set and 0.65, 0.609, and 0.539 in the test set, respectively, and 0.69, 0.771, and 0.731 in the full set, respectively (Figures 3C, F, I). With the median expression as the grouped cut-off, the Kaplan-Meier survival curve of each lncRNA in m6A-LPS was shown in Figure 3J. The m6A-LPS prognostic model performed well in different subgroups of clinicopathological characteristics. Overall survival of patients with high m6A-LPS risk scores and low m6A-LPS risk scores differed significantly among age (either ≤ 60 or >60 group), gender (either female or male group), stage (either stage I + II or stage III + IV group), TMN staging (T1+T2, T3+T4, M0, M1, N0+N1 or N2+N3 group) subgroups, and grade3 subgroups (Figures 4A–C).

### Construction of m6A-LPS containing nomogram

**FIGURE 5:**
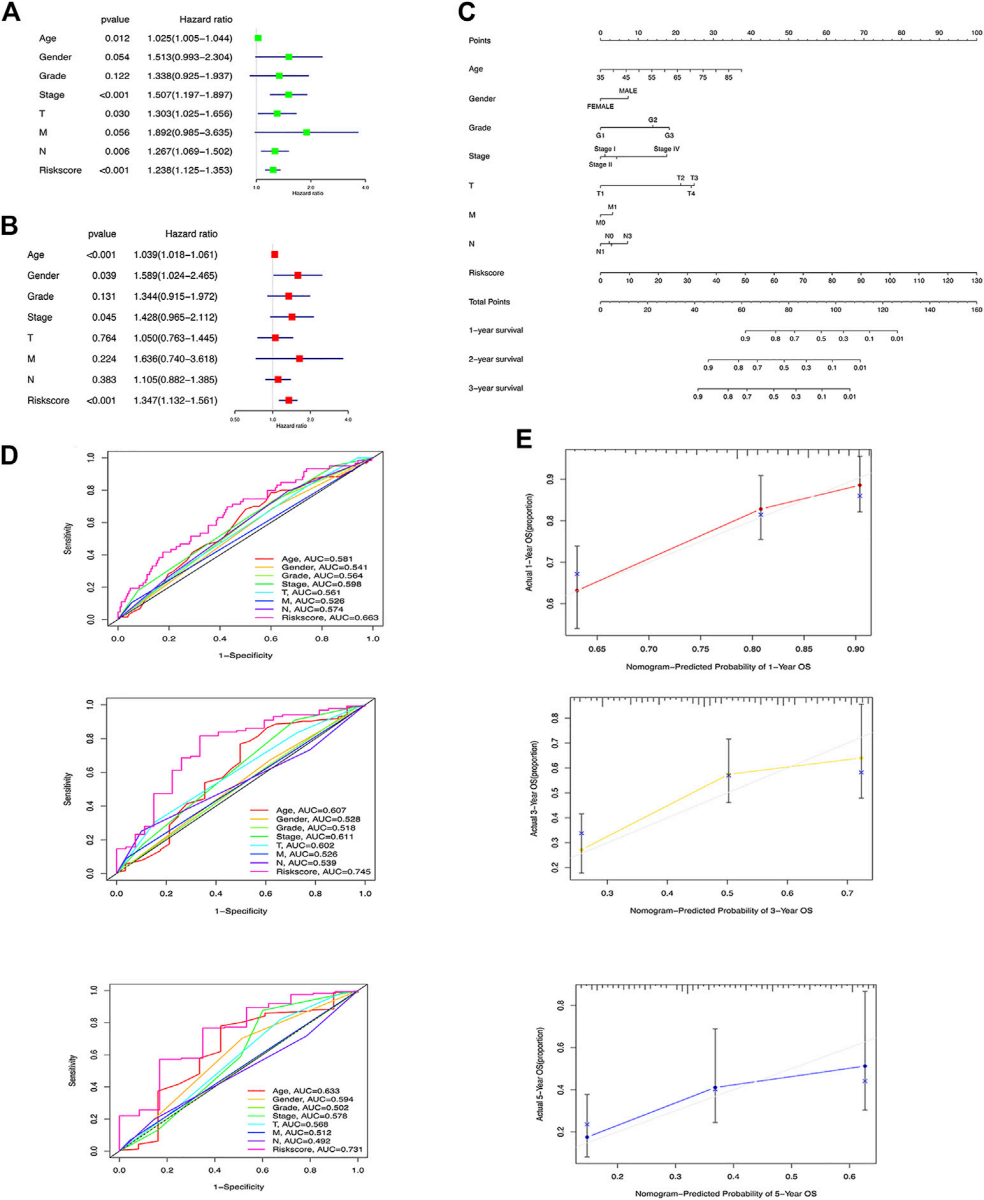
**(A)** Univariate and **(B)** multivariate Cox regression analyses on m6A-LPS risk score and age, sex, grade, stage, and TMN classification; **(C)** ROC curve of m6A-LPS for predicting 1/3/5-year overall survival of GC patients compared with age, gender, grade, stage, T,N, and M; **(D)** Nomograms constructed with m6A-LPS risk scores and age, sex, grade, stage, and TMN classifications for predicting the 1-, 3-, and 5-year survival rates of GC patients; **(E)** Calibration chart of nomogram for predicting 1/3/5 years survival.

To understand whether m6A-LPS is an independent risk factor for prognosis of GC patients, we performed univariate and multivariate Cox regression analyses on m6A-LPS risk score and age, sex, grade, stage, and TMN classification. The results showed that the m6A-LPS risk score and age, gender, and stage were independent risk factors for the prognosis of GC patients (Figures 5A, B). A nomogram was constructed with the m6A-LPS risk score and age, sex, grade, stage, and TMN classification for predicting 1-, 3-, and 5-year survival in GC patients (Figure 5C). As shown in Figure 5D, the strongest contributors to the nomogram prediction score were the risk score and age, with higher total scores associated with lower overall survival. Subsequently, we evaluated the identification and calibration performance of the nomogram by applying a calibration graph with a bootstrap of 1000 resamples. The 1-year, 3-year and 5-year survival rates predicted by the nomogram were compared with the actual survival rates, which were very close to the actual probabilities (Figure 5E).

### M6A-LPS function analysis

**FIGURE 6:**
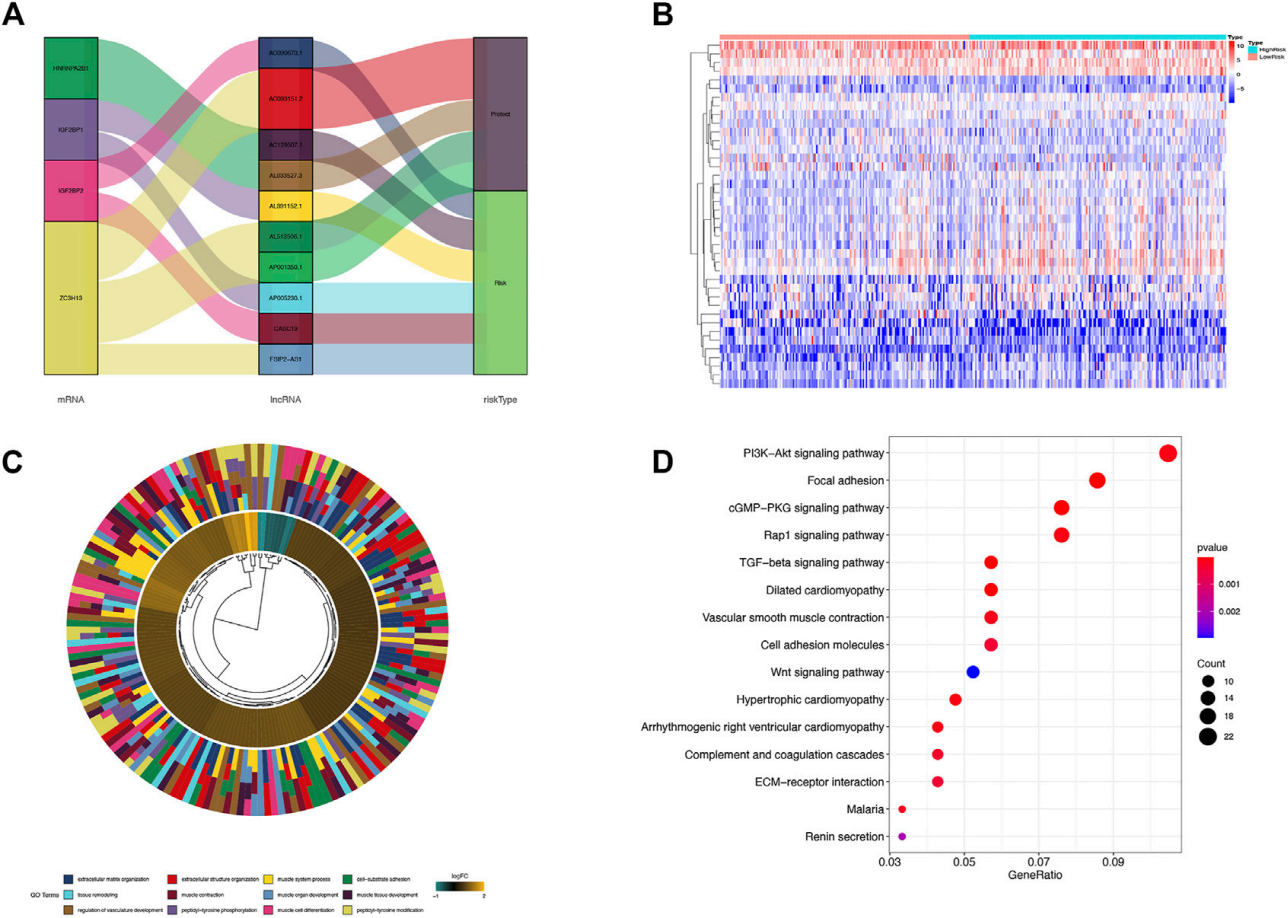
**(A)** Sankey diagram of the relationship among each lncRNA in m6A-LPS, m6A-related genes and survival risk types; **(B)** Heatmap of differentially expressed genes between the high and low risk score groups; **(C)** Gene ontology enrichment analysis of differentially expressed genes between high and low risk score groups; **(D)** KEGG pathway enrichment analysis of differentially expressed genes between high and low risk score groups.

Each lncRNA in m6A-LPS was associated with which m6A gene and what risk type it belongs to was shown in Figure 6A. Of the 11 lncRNAs in m6A-LPS, 5 were correlated with ZC3H13, 2 with IGF2BP2, 2 with IGF2BP1, 2 with HNRNPA2B1, 4 were protective factors, and 7 were risk factors. Differential expression analysis was performed for the high-risk and low-risk subgroups, and a total of 600 differentially expressed genes were obtained. The heatmap of the top 40 differentially expression genes were shown in Figure 6B.

GO function and KEGG signaling pathway enrichment analysis were performed on differentially expressed genes. The differentially expressed genes were significantly enriched in extracellular matrix/structure organization, cell-substrate adhesion, muscle cell differentiation, muscle contraction, muscle organ/tissue development, muscle system process, peptidyl-tyrosine modification/phosphorylation, regulation of vasculature development, and tissue remodeling GO entries and PI3K-Akt signaling pathway, Focal adhesion, cGMP-PKG signaling pathway, Rap 1 signaling pathway, TGF-beta signaling pathway, and Wnt signaling pathway (Figure 6C). The significantly enriched cancer-related signaling pathways were mainly PI3K-AKT signaling pathway, Focal adhesion, cGMP-PKG signaling pathway, Rap1 signaling pathway, TGF-beta signaling pathway, Cell adhesion molecules, Wnt signaling pathway, etc., (Figure 6D).

### Construction of ceRNA network and functional enrichment analysis

**FIGURE 7:**
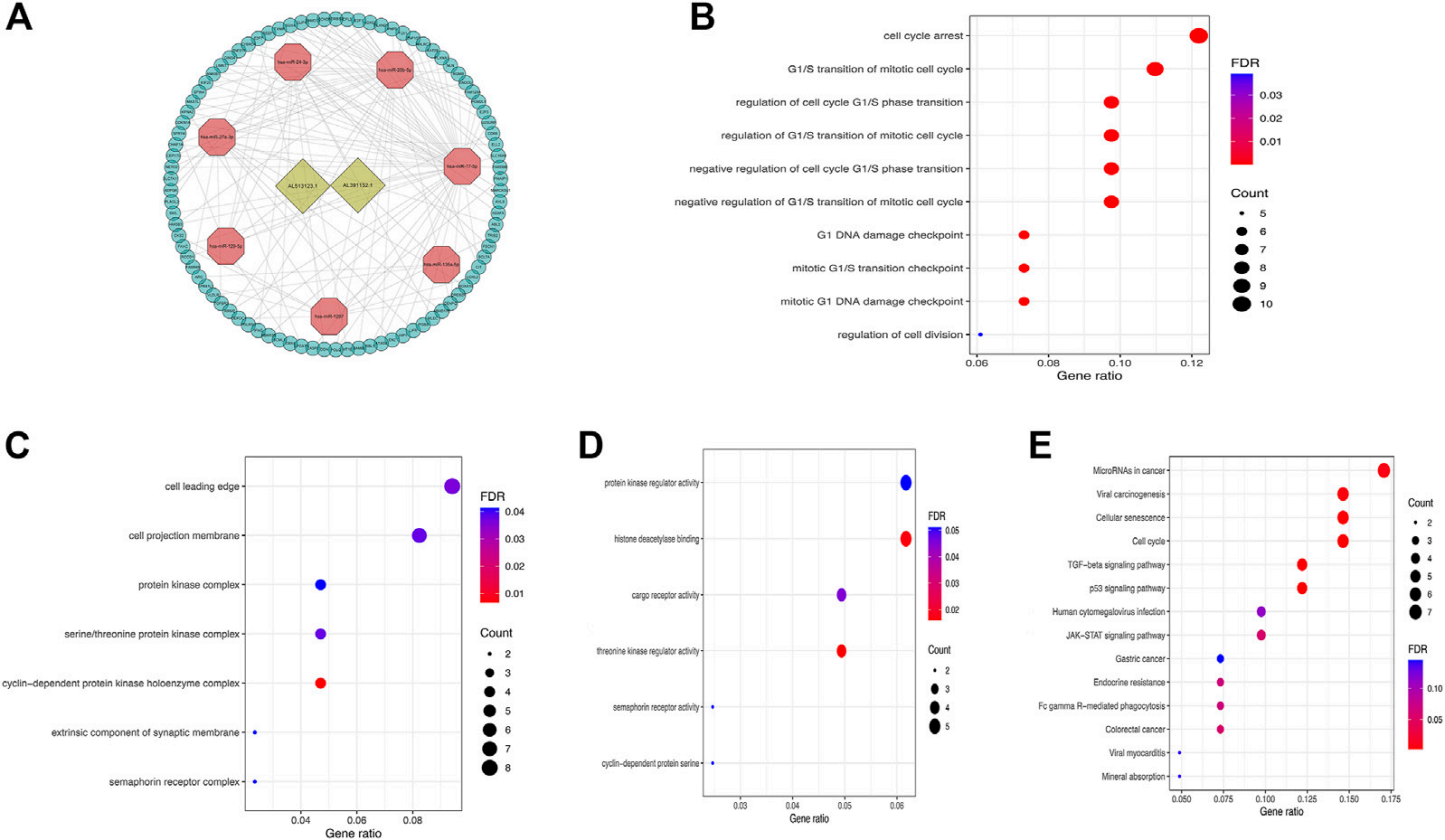
**(A)** The ceRNA network diagram of lncRNAs in m6A-LPS and their target miRNAs and mRNAs; **(B–E)** Biological process**(B)**, cellular components**(C)**, molecular functions**(D)** in GO and KEGG pathway **(E)** enrichment analysis of target mRNAs of miRNAs that interact with lncRNAs.

In order to understand how these m6A-related lncRNAs influence mRNA expression through sponging miRNAs, we found 2 lncRNAs (AL512506.1 and AL391152.1) and 7 miRNAs that interact with them by searching the miRcode database. According to these 7 miRNAs, 90 target mRNAs were obtained by searching in miRTarBase, miRDB, and TargetScan databases. The constructed ceRNA network diagram was presented in Figure 7A. To understand the biological functions of these target mRNAs in tumor microenvironment, we performed functional enrichment analysis on 90 target mRNAs. The results revealed that the target mRNAs were mainly enriched in biological processes such as cell cycle, cell division and signaling pathways such as cancer-related microRNAs, TGF-beta, p53, JAK-STAT, and GC (Figures 7B–E).

### lncRNA AL391152.1 regulates the expression of cyclins

**FIGURE 8:**
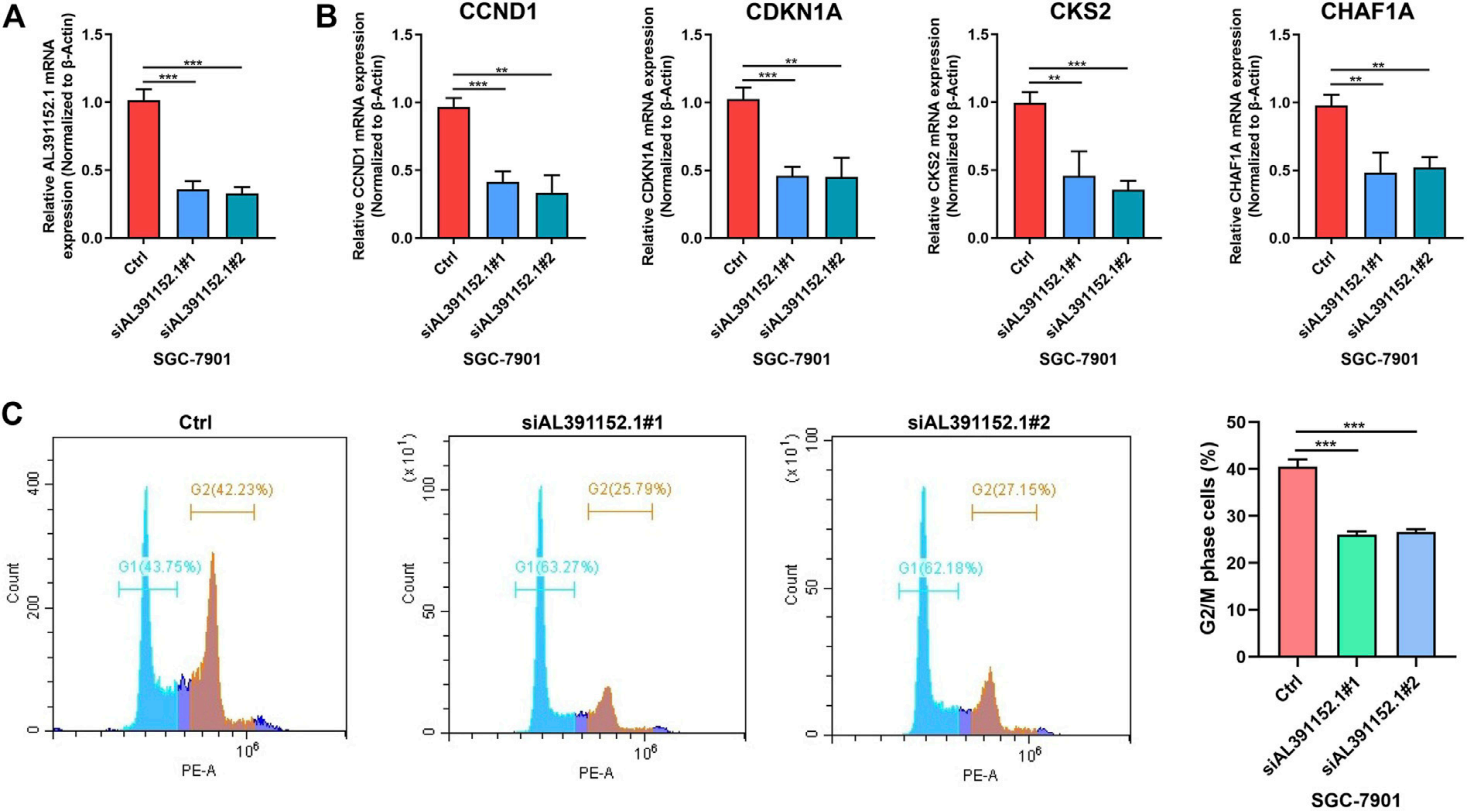
**(A)** qRT-PCR analysis of AL391152.1 expressions in SGC7901 cells after siRNA knock down of AL391152.1 or NC. **(B)** qRT-PCR analysis of CCND1, CDKN1A, CKS2 and CHAF1A expressions in SGC7901 cells after siRNA knock down of AL391152.1 or NC. **(C)** Cell cycle of SGC7901 was evaluated by flow cytometry and G2/S phase cells were quantified.

Based on our previous findings of m6A-related lncRNA AL391152.1 and its correlation with cell cycle, the relationship of AL391152.1 with cyclins were verified. The effect of silencing AL391152.1 expression in gastric cancer cell lines SGC-7901 were detected (Figure 8A). qRT-PCR demonstrated that depletion of AL391152.1 consistently resulted in reduction of CCND1, CDKN1A, CKS2 and CHAF1A mRNA levels in GC cells (Figure 8B). Flow cytometry confirmed the ability of AL391152.1 to control G2/M process (Figure 8C).

## Discussion

Currently, survival prediction for gastric cancer is mainly based on clinical or pathological TNM staging of prognosis developed by the American Joint Committee on Cancer. However, this staging method is primarily on the basis of anatomical location, and tumours in the same anatomical location may respond to treatment in very different ways, most likely due to heterogeneity at the molecular level. Therefore, the construction of prognostic models basing on genetic and clinicopathological features may better improve the prediction of survival in patients with gastric cancer.

In this study, we constructed m6A-related lncRNA prognostic models based on TCGA’s GC dataset by applying bioinformatics and machine learning methods. A total of 18 prognostic m6A-related lncRNAs were screened, 11 of which were used to construct the m6A-LPS prognostic model by LASSO regression analysis to calculate the risk scores. The results indicated that the m6A-LPS prognostic model performed well in predicting survival in both all and each clinicopathological subgroup of GC patients. The m6A-LPS risk score was confirmed by univariate and multifactorial COX regression as an independent risk factor for survival in patients with GC. Further, we constructed the nomogram by combining the m6A-LPS risk score together with common clinicopathological features, which also has good performance in predicting survival of GC patients.

The 11 lncRNAs incorporated in m6A-LPS were mainly associated with the m6A “Readers” genes HNRNPA2B1, IGF2BP1, IGF2BP1 and m6A “Writers” gene ZC3H13. These m6A genes have been shown to be associated with GC in several previous studies. The expression of hnRNPA2B1 protein was significantly higher in GC tissues compared to the para-carcinoma tissue (Dai et al., 2016). The expression of IGF2BP1 and IGF2BP2 mRNA was significantly increased in GC tissues, and the single nucleotide polymorphism locus rs9906944C > T located in IGF2BP1 gene was significantly correlated with reduced risk of GC (Wang et al., 2021). The ZC3H13 gene was the most frequently mutated of the m6A-related genes in GC, and the expression was significantly higher in GC tissues than in normal tissues (Zhang B. et al., 2020). However, the relationship between these m6A-related gene disorders and lncRNA in GC is unclear.

Among the prognostic m6A-related lncRNAs identified in this study, four were protective factors and seven were risk factors in GC. Among them, CASC19 upregulation has been reported to be associated with poor prognosis in GC and was an independent prognostic factor for overall survival, while silencing CASC19 inhibits proliferation and migration of GC cell (Wang et al., 2019). The protective factor AP001350.1 gene, a novel lncRNA antisense to ZFP91, was not yet clear about its role in GC, but high expression of ZFP91 promotes proliferation and invasion of gastric cancer (Peng et al., 2018; Tang et al., 2020). The AP001350.1-ZFP91 axis may be a potential mechanism for the development of GC. Similarly to AL033527.3, although there was no direct study of this lncRNA in gastric cancer, its possible antisense regulatory target BMP8B is associated with lymph node metastasis, liver metastasis and peritoneal dissemination in GC (Mima et al., 2013). The role of AP001350.1-BMP8B axis on GC was worth investigating. AC129507.1, also known as RPH3AL-AS1, was an antisense lncRNA for the RPH3AL gene, which was a well-known oncogene (Smith et al., 1999). This may explain how AC129507.1 promotes the progression of GC, requiring in-depth mechanistic studies. Unfortunately, the relationship between some of the lncRNAs in m6A-LPS and the development of GC or even cancer is still unclear, much less the interaction with m6A that has been studied.

To understand the functions that may be regulated by these m6A-related lncRNAs associated with prognosis of GC, we performed gene ontology and KEGG signaling pathway enrichment analysis on differentially expressed genes in the high and low-m6A-LPS risk score groups, enriching in a number of biological processes and signaling pathways associated with GC progression. Moreover, by constructing the ceRNA network, we found 7 miRNAs that may have interactions with lncRNAs, as well as 90 miRNA target mRNAs. Some miRNAs in the ceRNA network, such as miR-1297, hsa-miR-17-5p, hsa-miR-20b-5p, hsa-miR-125a-5p and hsa-miR-27a-3p, have been found to be associated with gastric cancer progression (Gao et al., 2018; Zhang X. et al., 2019; Chen et al., 2019; Chen et al., 2020; Jang et al., 2020; Liang et al., 2021). The m6A-related lncRNAs AL391152.1 and AL513123.1 may regulate the development or progression of gastric cancer by acting as a “sponge” for these miRNAs. Functional enrichment analysis of 90 target mRNAs further revealed possible regulatory mechanisms of m6A-related lncRNAs on GC progression. Our study testified that m6A-related lncRNA AL391152.1 regulates the expression of cell cycle progression related genes CCND1, CDKN1A, CKS2 and CHAF1A. CCND1 gene codes for the critical regulatory subunit of the enzyme responsible for phosphorylation and subsequent inactivation of the RB protein, leading to the cell cycle progression from G1 to S phase (Malumbres and Barbacid, 2001). CDKN1A acts simultaneously as a sensor and an effector of many anti-proliferative signals, and mediates cell cycle progress in tumorigenic milieu (Abbas and Dutta, 2009). CKS2 affect cell cycle progression by shortening of the cell cycle, increased replication fork velocity (Frontini et al., 2012). CHAF1A plays a central role in promoting cell cycle progression and proliferation (Tao et al., 2021). These clues may contribute to understanding the regulatory mechanisms of these m6A genes and related lncRNAs on GC development and progression.

In conclusion, a prognostic model of 11 m6A-associated lncRNA was constructed in this study, and a nomogram was constructed in combination with clinicopathological features, which performed well in predicting overall survival in the TCGA GC dataset. And the differentially expressed mRNAs in high- and low-risk score groups and the target mRNA of ceRNA network were performed function prediction to provide clues to reveal the mechanism of m6A-related lncRNAs in gastric cancer development and progression.

## Data Availability

The original contributions presented in the study are included in the article/Supplementary Material, further inquiries can be directed to the corresponding authors.

## References

[B1] Abbas T. Dutta A. (2009). p21 in cancer: intricate networks and multiple activities. Nat. Rev. Cancer 9 (6), 400–414. 10.1038/nrc2657 19440234 PMC2722839

[B2] Ajani J. A. D'Amico T. A. Almhanna K. Bentrem D. J. Chao J. Das P. (2016). Gastric cancer, version 3.2016, NCCN clinical practice guidelines in oncology. J. Natl. Compr. Canc Netw. 14 (10), 1286–1312. 10.6004/jnccn.2016.0137 27697982

[B3] Bayoumi A. S. Sayed A. Broskova Z. Teoh J. P. Wilson J. Su H. (2016). Crosstalk between long noncoding RNAs and MicroRNAs in health and disease. Int. J. Mol. Sci. 17 (3), 356. 10.3390/ijms17030356 26978351 PMC4813217

[B4] Chen H. Pan D. Yang Z. Li L. (2019). Integrated analysis and knockdown of RAB23 indicate the role of RAB23 in gastric adenocarcinoma. Ann. Transl. Med. 7 (23), 745. 10.21037/atm.2019.11.130 32042761 PMC6990024

[B5] Chen J. Chen J. G. Sun B. Wu J. H. Du C. Y. (2020). Integrative analysis of immune microenvironment-related CeRNA regulatory axis in gastric cancer. Math. Biosci. Eng. 17 (4), 3953–3971. 10.3934/mbe.2020219 32987562

[B6] Dai D. Wang H. Zhu L. Jin H. Wang X. (2018). N6-methyladenosine links RNA metabolism to cancer progression. Cell. Death Dis. 9 (2), 124. 10.1038/s41419-017-0129-x 29374143 PMC5833385

[B7] Dai P. Wang Q. Wang W. Jing R. Wang F. Azadzoi K. M. (2016). Unraveling molecular differences of gastric cancer by label-free quantitative proteomics analysis. Int. J. Mol. Sci. 17 (1), 69. 10.3390/ijms17010069 26805816 PMC4730314

[B8] Frontini M. Kukalev A. Leo E. Ng Y. M. Cervantes M. Cheng C. W. (2012). The CDK subunit CKS2 counteracts CKS1 to control cyclin A/CDK2 activity in maintaining replicative fidelity and neurodevelopment. Dev. Cell. 23 (2), 356–370. 10.1016/j.devcel.2012.06.018 22898779 PMC3898080

[B9] Gao W. Cao Y. Guo P. Bao X. Zhu H. Zheng J. (2018). Downregulation of MiR-1297 predicts poor prognosis and enhances gastric cancer cell growth by targeting CREB1. Biomed. Pharmacother. 105, 413–419. 10.1016/j.biopha.2018.05.094 29870889

[B10] He H. Wu W. Sun Z. Chai L. (2019). MiR-4429 prevented gastric cancer progression through targeting METTL3 to inhibit m(6)A-caused stabilization of SEC62. Biochem. Biophys. Res. Commun. 517 (4), 581–587. 10.1016/j.bbrc.2019.07.058 31395342

[B11] Jang M. G. Ko H. C. Kim S. J. (2020). Effects of p-coumaric acid on microRNA expression profiles in SNU-16 human gastric cancer cells. Genes. Genomics 42 (7), 817–825. 10.1007/s13258-020-00944-6 32462517

[B12] Liang Y. Zhao Y. Li L. Wei H. Huang T. Zhang H. (2021). MicroRNA profiles in five pairs of early gastric cancer tissues and adjacent non-cancerous tissues. Oncol. Lett. 22 (2), 595. 10.3892/ol.2021.12856 34149906 PMC8200934

[B13] Linder B. Grozhik A. V. Olarerin-George A. O. Meydan C. Mason C. E. Jaffrey S. R. (2015). Single-nucleotide-resolution mapping of m6A and m6Am throughout the transcriptome. Nat. Methods 12 (8), 767–772. 10.1038/nmeth.3453 26121403 PMC4487409

[B14] Malumbres M. Barbacid M. (2001). To cycle or not to cycle: A critical decision in cancer. Nat. Rev. Cancer 1 (3), 222–231. 10.1038/35106065 11902577

[B15] Meyer K. D. Saletore Y. Zumbo P. Elemento O. Mason C. E. Jaffrey S. R. (2012). Comprehensive analysis of mRNA methylation reveals enrichment in 3' UTRs and near stop codons. Cell. 149 (7), 1635–1646. 10.1016/j.cell.2012.05.003 22608085 PMC3383396

[B16] Mima K. Fukagawa T. Kurashige J. Takano Y. Uchi R. Ueo H. (2013). Gene expression of bone morphogenic protein 8B in the primary site, peripheral blood and bone marrow of patients with gastric cancer. Oncol. Lett. 6 (2), 387–392. 10.3892/ol.2013.1392 24137334 PMC3788827

[B17] Peng Y. Shen X. Jiang H. Chen Z. Wu J. Zhu Y. (2018). miR-188-5p suppresses gastric cancer cell proliferation and invasion via targeting ZFP91. Oncol. Res. 27 (1), 65–71. 10.3727/096504018X15191223015016 29471891 PMC7848256

[B18] Ritchie M. E. Phipson B. Wu D. Hu Y. Law C. W. Shi W. (2015). Limma powers differential expression analyses for RNA-sequencing and microarray studies. Nucleic Acids Res. 43 (7), e47. 10.1093/nar/gkv007 25605792 PMC4402510

[B19] Sasahara M. Kanda M. Kodera Y. (2021). Update on molecular biomarkers for diagnosis and prediction of prognosis and treatment responses in gastric cancer. Histol. Histopathol. 36 (8), 817–832. 10.14670/HH-18-326 33719028

[B20] Smith J. S. Tachibana I. Allen C. Chiappa S. A. Lee H. K. McIver B. (1999). Cloning of a human ortholog (RPH3AL) of (RNO)Rph3al from a candidate 17p13.3 medulloblastoma tumor suppressor locus. Genomics 59 (1), 97–101. 10.1006/geno.1999.5864 10395805

[B21] Su Y. Huang J. Hu J. (2019). m^6^A RNA methylation regulators contribute to malignant progression and have clinical prognostic impact in gastric cancer. Front. Oncol. 9, 1038. 10.3389/fonc.2019.01038 31681576 PMC6813557

[B22] Sung H. Ferlay J. Siegel R. L. Laversanne M. Soerjomataram I. Jemal A. (2021). Global cancer statistics 2020: GLOBOCAN estimates of incidence and mortality worldwide for 36 cancers in 185 countries. CA Cancer J. Clin. 71 (3), 209–249. 10.3322/caac.21660 33538338

[B23] Tang D. E. Dai Y. Xu Y. Lin L. W. Liu D. Z. Hong X. P. (2020). The ubiquitinase ZFP91 promotes tumor cell survival and confers chemoresistance through FOXA1 destabilization. Carcinogenesis 41 (1), 56–66. 10.1093/carcin/bgz085 31046116

[B24] Tao L. Moreno-Smith M. Ibarra-Garcia-Padilla R. Milazzo G. Drolet N. A. Hernandez B. E. (2021). CHAF1A blocks neuronal differentiation and promotes neuroblastoma oncogenesis via metabolic reprogramming. Adv. Sci. (Weinh). 8 (19), e2005047. 10.1002/advs.202005047 34365742 PMC8498874

[B25] Wang Q. Chen C. Ding Q. Zhao Y. Wang Z. Chen J. (2020). METTL3-mediated m(6)A modification of HDGF mRNA promotes gastric cancer progression and has prognostic significance. Gut 69 (7), 1193–1205. 10.1136/gutjnl-2019-319639 31582403

[B26] Wang W. J. Guo C. A. Li R. Xu Z. P. Yu J. P. Ye Y. (2019). Long non-coding RNA CASC19 is associated with the progression and prognosis of advanced gastric cancer. Aging (Albany NY) 11 (15), 5829–5847. 10.18632/aging.102190 31422382 PMC6710062

[B27] Wang X. Guan D. Wang D. Liu H. Wu Y. Gong W. (2021). Genetic variants in m(6)A regulators are associated with gastric cancer risk. Arch. Toxicol. 95 (3), 1081–1088. 10.1007/s00204-020-02958-1 33398416

[B28] Yang D. D. Chen Z. H. Yu K. Lu J. H. Wu Q. N. Wang Y. (2020). METTL3 promotes the progression of gastric cancer via targeting the MYC pathway. Front. Oncol. 10, 115. 10.3389/fonc.2020.00115 32175271 PMC7054453

[B29] Yu G. Wang L. G. Han Y. He Q. Y. (2012). clusterProfiler: an R package for comparing biological themes among gene clusters. OMICS 16 (5), 284–287. 10.1089/omi.2011.0118 22455463 PMC3339379

[B30] Yue B. Song C. Yang L. Cui R. Cheng X. Zhang Z. (2019). METTL3-mediated N6-methyladenosine modification is critical for epithelial-mesenchymal transition and metastasis of gastric cancer. Mol. Cancer 18 (1), 142. 10.1186/s12943-019-1065-4 31607270 PMC6790244

[B31] Zhang H. Shi X. Huang T. Zhao X. Chen W. Gu N. (2020a). Dynamic landscape and evolution of m6A methylation in human. Nucleic Acids Res. 48 (11), 6251–6264. 10.1093/nar/gkaa347 32406913 PMC7293016

[B32] Zhang B. Wu Q. Li B. Wang D. Wang L. Zhou Y. L. (2020b). m(6 A regulator-mediated methylation modification patterns and tumor microenvironment infiltration characterization in gastric cancer. Mol. Cancer 19 (1), 53. 10.1186/s12943-020-01170-0 32164750 PMC7066851

[B33] Zhang J. Guo S. Piao H. Y. Wang Y. Wu Y. Meng X. Y. (2019). ALKBH5 promotes invasion and metastasis of gastric cancer by decreasing methylation of the lncRNA NEAT1. J. Physiol. Biochem. 75 (3), 379–389. 10.1007/s13105-019-00690-8 31290116 PMC6728298

[B34] Zhang X. Zhang M. Guo Q. Hu X. Zhao Z. Ni L. (2019). MicroRNA-1297 inhibits proliferation and promotes apoptosis in gastric cancer cells by downregulating CDC6 expression. Anticancer Drugs 30 (8), 803–811. 10.1097/CAD.0000000000000776 31419217

[B35] Zhao B. S. Roundtree I. A. He C. (2017). Post-transcriptional gene regulation by mRNA modifications. Nat. Rev. Mol. Cell. Biol. 18 (1), 31–42. 10.1038/nrm.2016.132 27808276 PMC5167638

[B36] Zhu L. Zhu Y. Han S. Chen M. Song P. Dai D. (2019). Impaired autophagic degradation of lncRNA ARHGAP5-AS1 promotes chemoresistance in gastric cancer. Cell. Death Dis. 10 (6), 383. 10.1038/s41419-019-1585-2 31097692 PMC6522595

